# Advancing toxicity prediction in CAR-T cell therapy

**DOI:** 10.3389/fimmu.2026.1934301

**Published:** 2026-10-20

**Authors:** Andrea Palasciano, Stefano Bonfanti, Stefano Crippa

**Affiliations:** 1 Department of Medicine, Division of Clinical and Translational Research, McGill University, Montreal, QC, Canada; 2 Lady Davis Institute for Medical Research and Segal Cancer Center, Jewish General Hospital, Montreal, QC, Canada; 3 Fred Hutchinson Cancer Center, University of Washington Medicine, Seattle, WA, United States; 4 Department of Medicine and Surgery, University of Milano-Bicocca, Monza, Italy

**Keywords:** CAR-T cell therapy, CRS, ICAHT, ICANS, prediction

## Abstract

Despite recent advances in the field, Chimeric Antigen Receptor T (CAR-T) cell toxicities remain a significant burden throughout both the early and late post-infusion periods, affecting patient outcomes and complicating clinical management. Several risk factors for these side effects have been identified and multiple predictive scores and models have been developed, focusing mainly on clinical and inflammatory parameters assessed before or after CAR-T cell infusion. However, the majority of these scores have been derived and validated in retrospective cohorts, with external validation available only for a few of them predicting only CRS, ICANS or ICAHT risk. Several newly described toxicities still lack any predictive tool, and even the existing scores for CRS and ICANS have not yet been validated across all CAR-T disease indications, limiting their generalizability. The CAR-HEMATOTOX (CAR-HT), initially developed for ICAHT risk prediction, remains the most extensively validated tool to date. Despite this, no predictive model has yet been prospectively validated within interventional trials randomizing patients according to predicted risk. This minireview aims to provide an up-to-date overview of currently validated toxicity prediction scores in CAR-T cell therapy, along with prediction tools under development, future research directions and their potential integration into routine clinical practice to guide risk-adapted patient management.

## Introduction

CAR-T cell therapy has emerged as a transformative treatment modality for several hematologic malignancies (1–3), showing promising results also in non-hematological disorders (4). This treatment modality is associated with a unique spectrum of toxicities (5), including cytokine release syndrome (CRS), immune effector cell-associated neurotoxicity syndrome (ICANS) and immune effector cell-associated hematotoxicity (ICAHT) (6). Recently described toxicities include immune effector cell-associated hemophagocytic lymphohistiocytosis-like syndrome (IEC-HS) (7), tumor inflammation-associated neurotoxicity (TIAN) (8), movement and neurocognitive treatment-emergent adverse events (MNTs) (9) and local immune effector cell-associated toxicity (LICATS) (10). The incidence and severity of these toxicities are strongly influenced by disease setting and CAR-T cell construct (11).

Advances in the understanding of the pathophysiological mechanisms underlying these immune-mediated side effects have enabled the development of increasingly effective mitigation strategies and the identification of modifiable risk factors, substantially reducing the incidence and severity of early adverse events (12). The management of severe or refractory, early and late toxicities, still represents a major area of ongoing investigation. Novel strategies, including prophylactic and pre-emptive interventions, are currently being evaluated (13).

To support risk stratification and clinical decision-making, multiple validated and emerging pre-infusion and peri-infusion scoring systems have been developed to predict CAR-T-related toxicities.

These models differ in methodology, predictive performance and clinical applicability, largely influenced by the characteristics of their validation cohorts, including disease type and CAR-T product. Of note, certain disease types may be more affected by the extent of marrow involvement and CAR-T constructs incorporating a CD 28 costimulatory domain have consistently reported to induce higher rates CRS/ICANS. Despite the heterogeneity and the bias of confounding factors, theses risk scoring systems collectively represent an important step towards personalized toxicity management.

A structured PubMed/MEDLINE search (April 2016–April 2026) was performed to identify all published predictive tools for CAR-T cell toxicities. Tools with both internal and external validation are summarized under “Currently Validated Predictive Scoring Systems”, whereas those lacking external validation to date are summarized under “Scores and Models Under Development”.

## Currently validated predictive scoring systems for CAR-T cell toxicities

Several risk factors for immune mediated toxicities post CAR-T cell therapy have been identified, primarily inflammatory markers, disease burden and bone marrow reserve (12). The most widely validated scores for predicting CRS and ICANS are the Endothelial Activation and Stress Index (EASIX) derived scores (EASIX, s-EASIX, m-EASIX and ferritin-based modifications). The EASIX score $\left(\frac{\text{lactate dehydrogenase}[\text{LDH};\text{ U}/\text{L}]\times \text{creatinine }[\text{mg}/\text{dL}]}{\text{platelets}[\text{PLTs};\;10^{9}\text{ cells}/\text{L}]}\right)$ is predictive of endothelial damage and correlates with outcomes after allogeneic hematopoietic cell transplantation (14). The proposed simplified EASIX (s-EASIX) excludes creatinine, while the modified EASIX (m-EASIX) replaces creatinine with CRP (mg/dL), measured at different time point (lymphodepletion, day –1, day +1 and day +3). EASIX scores were originally developed and tested in a retrospective analysis involving patients affected by diffuse large B cell lymphoma (DLBCL) who were treated with axicabtagene ciloleucel (axi-cel) or tisagenlecleucel (tisa-cel) and B-acute lymphoblastic leukemia (ALL) patients who received anti-CD19-28z CAR-T cells (15). The EASIX/s-EASIX/m-EASIX scores were confirmed to be good predictors for severe CRS at each time point, while only day +3 assessments were found to correlate with severe ICANS onset (15). EASIX variations incorporating ferritin (EASIX-F) or CRP and ferritin (EASIX-FC) were able to predict respectively CRS ≥ grade 2 and ICANS ≥ grade 2 in a single-center retrospective analysis that evaluated 171 patients with large B cell lymphomas (LBCL) who received axi-cel (16). EASIX, m-EASIX, s-EASIX, and EASIX-FC were externally validated in a population-based cohort study, including 154 patients with relapsed/refractory LBCL who received axi-cel after ≥2 lines of systemic therapy (17).

The m-EASIX score was also recently validated in a cohort of non-Hodgkin lymphoma (NHL) and multiple myeloma (MM) patients receiving, respectively, anti-CD19 and anti-BCMA CAR-T cell therapy, where it proved to be a stronger predictor of severe toxicities and ICU admissions than EASIX. Furthermore, m-EASIX could accurately distinguish CRS from sepsis at symptom onset (18).

Two additional prognostic scoring systems (PSS) were derived from the DESCAR-T registry, which included 925 patients with LBCL treated with axi-cel or tisa-cel. The CRS-PSS incorporated bulky disease, platelet count <150 G/L, CRP >30 mg/L, and no bridging therapy or SD/PD after bridging, with a score >2 predicting grade ≥3 CRS. Similarly, the ICANS-PSS incorporated female sex, platelet count <150 x 10^9^/L, axi-cel use, and no bridging therapy or SD/PD after bridging, with a score >2 predicting grade ≥3 ICANS. Both scores were externally validated in UK and Spanish cohorts (19).

Cytokine-based models have also shown promise in predicting CRS. To date, the only externally validated model is a multi-cytokine nomogram developed by Zhou et al., derived from a cohort of 87 MM patients and then externally validated in 2 separate cohorts (59 MM and 68 ALL/NHL patients). Built on five cytokines (CX3CL1, GZMB, IL-4, IL-6, and PDGF-AA), measured on days 1–2 post-infusion, the nomogram achieved strong discriminative performance for severe CRS. Its reliance on cytokine measurements not routinely available in clinical practice, however, limits its feasibility for widespread use (20).

ICAHT is another common toxicity associated with CAR-T cell therapy, which is graded according to the depth and duration of neutropenia. The CAR-HEMATOTOX (CAR-HT) score is the only externally validated tool for predicting ICAHT. It incorporates hematopoietic reserve (platelets, hemoglobin, and absolute neutrophil count) and baseline inflammatory markers (CRP and ferritin). Patients are stratified as high-risk (score ≥2) or low-risk (score 0–1). CAR-HT was originally developed by Rejeski et al. in a training cohort of 58 LBCL patients and validated in two independent external cohorts, one European (n = 91) and one US (n = 109) (21). This has been further externally validated across multiple disease types and settings including patients with LBCL (21), mantle cell lymphoma (MCL) (22) and MM (23).

Beyond ICAHT itself, the CAR-HT score has been shown to predict several additional outcomes, extending its utility well beyond cytopenia alone. High CAR-HT scores (≥2) have been consistently associated with increased infection rates (24), especially bacterial, across LBCL, MCL, and MM cohorts. Validation studies in both BCMA-directed CAR-T for MM (23)and anti-CD19 CAR-T for MCL (22) demonstrated that high CAR-HT scores were associated with increased non-relapse mortality and further studies have confirmed that a high CAR-HT score can independently predict inferior overall survival (OS) and progression free survival (PFS) (25, 24).

An adapted version of the score for ALL, the ALL-HEMATOTOX (ALL-HT), has also been developed and validated (26). This adaptation was prompted by the limited discriminatory power of CAR-HT in ALL: because most patients have elevated ferritin baseline levels, the score fails to meaningfully stratify patients into actionable risk groups for hematotoxicity management. The ALL-HT addresses this by replacing ferritin with bone marrow disease burden, which correlates strongly with post-infusion neutropenia in ALL (r = 0.64, p <.0001) (26).

**Table 1** shows the currently validated tools for CRS, ICANS, and ICAHT prediction, including the time points at which they are assessed and both derivation and validation cohorts characteristics, such as CAR-T cell construct and disease type. Among these, the CAR-HT score stands out as the most extensively validated across different disease types. Among these, the CAR-HT score has undergone the most extensive validation across multiple disease settings. Nevertheless, no predictive model has yet been shown to improve clinical outcomes when applied prospectively to guide risk-adapted interventions, underscoring an important unmet need.

**Table 1:** Validated scores for CRS/ICANS/ICAHT prediction – In this table are shown the main scores validated for CRS/ICANS/ICAHT prediction, including the specific type of toxicity they predict, the population in which they were derived and externally validated.

Score	Tox	Timing	Variables	Derivation and internal validation				External validation			
				Disease type	CAR-T product	AUC of the ROC curve (%)		Disease type	CAR-T product	AUC of the ROC curve (%)	Ref
							Ref				
*EASIX*	CRS ICANS	LD D-1 D +1 D +3	LDH Crea PLT	B-ALL + DLBCL (n=118)	CD1928z Axi-cel Tisa-cel	Severe CRS: 77.3^a^, 71.8^b^, 72.4^c^, 79.7^d^ Severe ICANS: 61.2^c^, 68.0^d^	(15)	LBCL (n=214)	Axi-cel Tisa-cel	Severe toxicity: 0.81^a^	(27)
								LBCL (n=154)	Axi-cel	Severe CRS: 54^e^, 55^f^, 55^g^ Severe ICANS: 57^e^, 62^f^, 61^g^	(17)
*s-EASIX*	CRS ICANS	LD D-1 D +1 D +3	LDH PLT	B-ALL + DLBCL (n=118)	CD1928z Axi-cel Tisa-cel	Severe CRS: 82.1^a^, 75.4^b^, 76.4^c^, 80.9^d^ Severe ICANS: 65.8^c^, 68.4^d^	(15)	LBCL (n=214)	Axi-cel Tisa-cel	Severe toxicity: 0.79^a^	(27)
								LBCL (n=154)	Axi-cel	Severe CRS: 56^e^, 55^f^, 57^g^ Severe ICANS: 58^e^, 61^f^,61^g^	(17)
*m-EASIX*	CRS ICANS	LD D-1 D +1 D +3	LDH CRP PLT	B-ALL + DLBCL (n=118)	CD1928z Axi-cel Tisa-cel	Severe CRS: 80.4^a^, 73.0^b^, 75.4^c^, 80.2^d^ Severe ICANS: 67.0^c^, 73.0^d^	(15)	LBCL (n=214)	Axi-cel Tisa-cel	Severe toxicity 0.74^a^	(27)
								LBCL (n=154)	Axi-cel	Severe CRS: 57^e^, 59^f^, 58^g^ Severe ICANS: 46^e^, 59^f^, 59^g^	(17)
								ALL+NHL+ MM (n=119)	Var-cel Cesni-cel Axi-cel Liso-cel	Severe toxicity: 71^b^, 73^c^	(18)
								LBCL (n=38)	Axi-cel Tisa-cel Brexu-cel	Severe CRS: 67^a^ Severe ICANS: Not predictive	(28)
*EASIX-FC*	ICANS	LD	LDH PLT Ferritin CRP	LBCL (n=171)	Axi-cel	Not reported	(16)	LBCL (n=154)	Axi-cel	Not reported	(17)
CRS-PSS	CRS	LD	Bulk (> 5cm) PLT <150 CRP>30mg/L No BT/failure	LBCL (n=925)	Axi-cel Tisa-cel	Severe CRS: 72^a^	(19)	LBCL (n=725)	Axi-cel Tisa-cel	Severe CRS: 61^a^	(19)
ICANS-PSS	ICANS	LD	Female PLT <150 No BT/failure Axi-cel	LBCL (n=925)	Axi-cel Tisa-cel	Severe ICANS: 75^a^	(19)	LBCL (n=760)	Axi-cel Tisa-cel	Severe ICANS: 71^a^	(19)
Cytokine Nomogram	CRS	D+1	CX3CL1 GZMB IL-4 IL-6 PDGF-AA	MM (n=87)	Anti-BCMA	Severe CRS: 88^c^	(20)	Cohort 1 MM (n=59)	Anti-BCMA	Severe CRS: 91^c^	(20)
								Cohort 2 ALL+NHL (n=68)	Anti-CD19	Severe CRS: 91^c^	(20)
CAR-HT*	ICAHT	LD	PLT ANC Hb CRP Ferritin	DLBCL (n=58 training cohort; n=91 EU validation; n=109 US validation)	Axi-cel Tisa-cel	Severe ICAHT in HT^high^: Training cohort 73 EU validation 77 US validation 91	(21)	MM (n=113)	Ide-cel Cilta-cel	Severe ICAHT in HT^high^: 82	(23)
								MCL (n=103)	Brexu-cel	Severe ICAHT in HT^high^: 84	(22)
								LBL (n=560)	Axi-cel Tisa-cel	Severe ICAHT in HT^high^: 62.5	(25)
								LBL (n=250)	Axi-cel Tisa-cel	Severe ICAHT in HT^high^: 73	(24)
								ALL adults (n=199)	Brexu-cel	Delayed ANC recovery: 65 Delayed PLT recovery: 64 G3+ infections: 85	(29)
ALL-HT	ICAHT	LD	PLT ANC Hb CRP BM disease	ALL children and AYA (n= 156 training cohort NCI; n=106 validation cohort MSK; n= 84 SCH)	CD19–22 bicistronic CD19–22 bivalent CD19/28z CD22/41BB	Severe ICAHT in HT^high^: NCI cohort 84 MSK cohort 89 SCH cohort 90	(26)	External validation provided in independent external cohorts within original study			

As statistical measure of score performance the area under the curve (AUC) of the receiver operating characteristic (ROC) curve was reported. LD= lymphodepletion, D-1 = 1 day prior to CAR-T cell infusion, D + 1 = 1 day following CAR-T cell infusion, D + 3 = 3 days following CAR-T cell infusion. ^a^for score measured at LD, ^b^for score measured at D-1, ^c^for score measured at D + 1, ^d^for score measured at D + 3, ^e^for score measured at baseline, ^f^for score measured at pre-LD, ^g^for score measured at infusion. PLT, platelet; CRP, C-reactive protein; LDH, lactate dehydrogenase; No BT/failure, no bridging therapy or stable or progressive disease (SD/PD) after bridging; ANC, absolute neutrophil count; Hb, haemoglobin; CRP, C-reactive protein; HT^high^, high risk group for hematotoxicity; * An online calculator from German Lymphoma Alliance is available for CAR-HT (https://www.german-lymphoma-alliance.de/Scores.html).

## Scores and models under development

Although several predictive models for CAR-T related toxicities have been validated, the search for additional scores and biomarkers remains ongoing. For CRS, ICANS and ICAHT, recent efforts have focused on refining prediction through models that incorporate novel biomarkers and clinical variables including also other non-hematologic parameters. For instance, the CRS-Pre-infusion Risk Evaluation score (CRS-PRE) identified impaired renal function as a predictor of severe CRS in patients with LBCL treated with axi-cel, lisocabtagene maraleucel (liso-cel), or tisa-cel, together with elevated LDH, CRP or ferritin. Patients classified as high risk experienced a higher cumulative incidence of any grade CRS (30). Nutritional status may also impact CAR-T cell treatment outcomes: the Controlling Nutritional Status (CONUT) score, for instance, has been demonstrated to affect the prognosis of patients undergoing CAR-T cell therapy for MM (31, 32). Both models are extremely promising given the use of reliable and easily accessible parameters, even if limited to a specific patient population.

The incorporation of inflammatory burden markers may further enhance the predictive accuracy of ICANS risk models. A recently developed model incorporating CRP, IL-6, and procalcitonin demonstrated accurate prediction of both ICANS occurrence and severity in patients treated with anti-CD19 or anti-BCMA CAR-T cells. Given the well-known relationship between ICANS and inflammation burden, this score could provide a reliable and easily available tool. Furthermore, it would allow a dynamic evaluation and a broad applicability (33). In a retrospective cohort of patients with DLBCL treated with anti-CD19 CAR-T cells, elevated IL-6 and IL-15 levels alongside a prior history of autoimmune disease were independently associated with an increased risk of ICANS. Based on this, the authors developed two cytokine-based prediction models: one for any-grade ICANS, incorporating CAR-T product (axi-cel vs. tisa-cel), time to CRS onset, IL-6 at day 3, and pre-infusion D-dimer, and one for grade 2–4 ICANS, based on number of prior therapies, grade ≥2 CRS, history of autoimmune disease, IL-15 at day 0, and GM-CSF (34). Nonetheless, the use of some non-routinely evaluated cytokines could be an obstacle to the clinical application of this score.

Machine learning techniques are increasingly being integrated into many aspects of healthcare and are particularly attractive for developing predictive models because they can capture complex relationships among multiple variables that may be missed by conventional statistics. An XGBoost (eXtreme Gradient Boosting) algorithm incorporating age, pre- and post-infusion temperature, ferritin, CRP, LDH, IL-6, fibrinogen, and platelet count was able to predict grade ≥3 ICANS before the onset of clinical symptoms, showing high discriminative performance in an internally validated cohort of patients with B-cell lymphoma receiving anti-CD19 CAR-T therapy (35). Nevertheless, issues related to model interpretability, generalizability, and the need for robust external validation remain important barriers to their routine clinical implementation.

Additional biomarkers have also emerged as potential predictors of ICANS. Among these, serum neurofilament light chain (NfL), a well-established marker of neuroaxonal injury in several neurological disorders (36), has shown promising predictive value. A recent retrospective study demonstrated that elevated NfL levels were independently associated with the development of grade 2–4 ICANS in patients with B-cell lymphoma receiving CD19-directed CAR-T cell therapy (37). These findings suggest that biomarkers reflecting pre-existing neurological vulnerability may improve ICANS risk stratification beyond currently available clinical prediction models. However, this marker has only been evaluated in LBLC patients and is not readily available in the routine clinical setting.

Further efforts to refine ICAHT prediction are ongoing. A comprehensive univariate analysis, conducted in a cohort of 691 patients (NHL, MCL, MM, and ALL), identified both pre-infusion factors (disease type [ALL vs. others] and pre-lymphodepletion absolute neutrophils count [ANC], platelet count, LDH, and ferritin) and post-infusion factors (early and peak levels of CRP, ferritin, IL-6, and D-dimer, as well as peak CRS grade and peak neurotoxicity grade) associated with severe early ICAHT (eICAHT). Based on these findings, two eICAHT Prediction Models (eIPMs) were developed: eIPM-Pre, incorporating pre-infusion factors alone, and eIPM-Post, incorporating both pre-infusion and early post-infusion factors. Both models showed excellent discriminative performance (AUC 0.87 and 0.88, respectively), though eIPMs have so far undergone only internal validation and have not yet been validated in a separate cohort. Notably, by incorporating dynamic inflammatory biomarker evaluation, eIPM suggests that tracking immune activation over time may add prognostic value for predicting severe early ICAHT (38).

Several recently described CAR-T cell associated toxicities still lack validated predictive tools for clinical use. IEC-HS remains an underexplored area in predictive risk stratification. No validated predictive score is currently available (39). Existing approaches rely on diagnostic rather than predictive criteria: the ASTCT consensus identified high ferritin, cytopenias, coagulopathy with hypofibrinogenemia, and transaminitis as key features associated with this complication (40). Research efforts are focused on identifying additional risk factors, including disease type (with the highest incidence reported in B-ALL), CAR-T cell product tisa-cel in DLBCL and ciltacabtagene autoleucel (cilta-cel) in MM and the occurrence of CRS (41). Distinct cytokine profiles, including elevated TNF, IL-18, and MCP-1 levels, as well as specific immune cell subsets such as non-classical monocytes and natural killer T (NKT) cells, have also been associated with IEC-HS (42). The H-Score has not been validated in the CAR-T cell setting. Moreover, its utility in IEC-HS may be limited by the substantial clinical and laboratory overlap with CRS (43).

Similarly, many recently described neurotoxicity syndromes lack validated predictive scores. A recent study (44) conducted in patients with primary CNS lymphoma treated with anti-CD19 CAR-T demonstrated that baseline MRI-measured CNS tumor volume is a significant predictor of TIAN development, an immune effector cell-mediated neurotoxicity syndrome occurring in patients with CNS cancers (45). Other neurotoxicity syndromes of interest are MNTs, which are characterized by Parkinson-disease-like symptoms (such as bradykinesia, rigidity, tremor and cognitive impairment) (9), and CNP, which is usually isolated and primarily involving VII cranial nerve, even if some progressive cases have been reported (46). Both are predominantly observed in MM patients treated with anti-BCMA CAR-T, especially cilta-cel (47, 48). Nonetheless few cases have been reported in B cell lymphomas (49, 50), and pediatric B-ALL (51) patients receiving anti-CD19 CAR-T cells. To date, there are no validated risk predicting tools, but several risk factors have been identified. A sub-analysis from the CARTITUDE-1 study identified high tumor burden, high grade CRS, any grade ICANS, high CAR-T cell expansion and persistence in peripheral blood and CSF as potential MNT anticipating factors (52). Absolute lymphocyte count (ALC) peak was found to be a statistically significant predictor of MNTs and CNP in cilta-cel-treated patients (53). Another retrospective analysis conducted on patients from CARTITUDE 1, 2 and 4 studies found a correlation between CNP frequency, higher peak concentrations of IL-10 and a lower number of prior lines of therapy, suggesting a role for T cell immune fitness in the onset of this neurotoxicity (54).

A newly described post-CAR-T toxicity, termed LICATS, has recently been reported in patients with autoimmune diseases receiving CD19-directed CAR-T cells. It is characterized by a local, organ-specific inflammatory reaction following CAR-T cell infusion, exclusively involving organs previously affected by the patient’s underlying autoimmune disease. In a recent observational study, all cases of LICATS occurred during B-cell aplasia, and grade ≥2 LICATS was associated with grade 2 CRS and tocilizumab use (10). Aside from disease burden, no additional risk factors have yet been identified. As novel CAR-T-related toxicities such as LICATS continue to emerge, this highlights the need for further characterization and the development of dedicated prediction strategies, scores, and models.

Growing evidence indicates that the gut microbiome significantly influences both the incidence and severity of CAR-T cell therapy toxicities, particularly cytokine release syndrome (CRS) and immune effector cell-associated neurotoxicity syndrome (ICANS). These data, while consistent across multiple cohorts, remain preliminary and largely observational (55). The most robust and clinically actionable finding is the association between prior broad-spectrum antibiotic exposure and worse CAR-T outcomes (55). Several studies have identified specific bacterial species linked to CAR-T toxicities (56, 57), which they mediate through their effects on systemic inflammation, short-chain fatty acid (SCFA) production, and immune homeostasis. Further research will hopefully help guide additional strategies for toxicity prevention.

**Table 2** recapitulates the main scores validated and under development for each toxicity.

**Table 2:** Toxicity predicting tools available – In this table are shown the validated predicting tools and the ones still under development for the more commonly described CAR-T cell related toxicities.

Toxicity	Toxicity predicting tools available					
	Validated	Ref	Under development (lacking external validation)			Ref
CRS	EASIX	(15)	CRS-PRE	Disease type	CAR-T product	
				LBCL (n=900)	Axi-cel Tisa-cel Liso-cel	(30)
	s-EASIX	(15)				
	m-EASIX	(15)				
	CRSS-PSS	(19)	CONUT	MM (n=300)	Anti-BCMA Anti-GPRC5D Anti BCMA/CD19 Anti BCMA/GPRC5D	(32)
	Cytokine nomogram	(20)				
ICANS	EASIX	(15)	Hidden Markov Model (HMM) to forecast ICANS	LBCL + MM (n=199)	Anti-CD19 Anti-BCMA	(33)
	s-EASIX	(15)				
	m-EASIX	(19)	Cytokine based prediction models	ALL + NHL (n=101)	Tisa-cel Axi-cel	(34)
	EASIX-FC	(16)				
	ICANS-PSS	(19)	XGBoost algorithm	NHL (n=175)	Axi-cel Brexu-cel	(35)
ICAHT	CAR-HT	(21)	eIPMs (only tool available at https://eipm.fredhutch.org)	NHL+ALL+MM (m=691)	Anti-CD19 (JCAR014^a^, Axi-cel, Liso-cel, Tisa-cel, Brexu-cel, JCAR021^b^); Anti-CD20 (investigational); Anti-BCMA (cilta-cel, ide-cel, investigational)	(38)
	ALL-HT	(26)				
IEC-HS	No risk predicting tools available.					(42)
TIAN	No validated risk predicting tools available. MRI-measured CNS tumor volume is the only know predictor.					(44)
MNTs	No validated risk predicting tools available, single individual risk factors identified: high tumor burden, high grade CRS, any grade ICANS and high CAR-T cell expansion, ALC peak.					(48, 52, 53)
CNP	No risk predicting tools available, single individual risk factors identified: ALC peak, IL-10 levels.					(48, 53, 54)
LICATS	No risk predicting tools available. Disease burden main risk factor.					(10)

For IEC-HS, TIAN, MNTs, CNP and LICATs no predicting tools are available and only few isolated predictors have been described. ^a^JCAR014, investigational CAR T-cell product containing 4-1BB costimulatory domain; ^b^JCAR021, investigational CAR T-cell product containing 4-1BB costimulatory domain.

## Implementation into clinical decision making

The substantial body of evidence accumulated over the past decade has profoundly shaped clinical practice worldwide, driving the use of CAR-T cell products into earlier lines of treatment and enabling the development of well-defined protocols for the prevention and management of the most clinically relevant toxicities. The lessons learned from clinical trials and real-world practice have charted a path of continuous improvement, not only in terms of efficacy through novel CAR-T cell designs, but also in terms of safety and toxicity prevention. As CAR-T cell therapy expands into non-oncological diseases, where tolerance for severe side effects is lower, understanding, classifying, and preventing CAR-T-related toxicities is now more critical than ever.

Among the validated scores for CAR-T cell toxicities prediction, the CAR-HT score is currently the only one incorporated into clinical guidelines. Specifically, CAR-HT score ≥2 contributes to high risk ICAHT categorization by NCCN guidelines with recommended granulocyte-colony stimulating factor (G-CSF) initiation at ≥2 days post infusion (58). The EHA/EBMT consensus similarly recommends antibacterial prophylaxis only in high ICAHT-risk patients once ANC falls below 500/µL, and suggests considering antifungal prophylaxis in cases of severe neutropenia with a high-risk profile (59). For cytopenias persisting beyond 21 days post-infusion with ≥1 risk factor, the NCCN recommends considering G-CSF for neutropenia, eltrombopag for prolonged thrombocytopenia, or a hematopoietic cell boost when cryopreserved stem cells are available (58).

None of the other validated scores or models discussed in the previous section has yet been incorporated into European or American guidelines, and their role in clinical practice therefore remains uncertain. At present, the main determinants of toxicity remain underlying diagnosis, CAR-T construct, disease burden, inflammatory burden, and marrow reserve, with adequate patient selection, bridging therapy to reduce disease burden, and prophylactic measures guided by pre-lymphodepletion assessment playing a defining role in risk mitigation.

## Conclusions

Several priorities emerge for the CAR-T related toxicities management field moving forward. First, existing predictive models for CRS and ICANS require further external validation across broader settings, including MM and non-oncological indications, while newly proposed scores not yet externally validated similarly await confirmation in independent cohorts. Second, dynamic models incorporating multiple time points along the CAR-T cell journey warrant further refinement, enabling more precise and timely tailoring of supportive measures.

Notably, all current CAR-T toxicity prediction scores have been derived exclusively from retrospective or registry-based analyses. No prospective trial has yet tested score-guided risk-adapted management strategies. A critical next step for the field would be prospective interventional trials randomizing patients according to predicted risk, for example, CAR-HEMATOTOX high versus low, to risk-adapted management strategies, such as early G-CSF, prophylactic anakinra, or intensified monitoring, versus standard care, with toxicity severity and non-relapse mortality as primary endpoints. Among existing tools, the CAR-HT score represents the strongest candidate for such trials, though its predictive value at earlier time points, prior to leukapheresis, remains to be established, which would be particularly valuable in enabling prophylactic collection of autologous CD34+ stem cells.

Finally, as CAR-T cell therapy indications continue to expand, it is now of paramount importance to characterize, develop and validate predictive models for the full spectrum of emerging toxicities, including TIAN, MNTs, CNP, and LICATS. Studies providing external validation of the scores and biomarkers discussed herein will be essential not only to refine clinical management of toxicities for which risk assessment tools already exist, but also to establish robust risk stratification frameworks for those that do not.
